# External Condensation of HFE 7000 and HFE 7100 Refrigerants in Shell and Tube Heat Exchangers

**DOI:** 10.3390/ma14226825

**Published:** 2021-11-12

**Authors:** Marcin Kruzel, Tadeusz Bohdal, Krzysztof Dutkowski

**Affiliations:** Power Engineering Department, Koszalin University of Technology, 75-453 Koszalin, Poland; tadeusz.bohdal@tu.koszalin.pl (T.B.); krzysztof.dutkowski@tu.koszalin.pl (K.D.)

**Keywords:** heat exchanger, condensation, heat transfer coefficient

## Abstract

The paper describes the results of experimental studies of media as an intermediary in heat exchange taking place in low volume conditions. Their properties predestine them both as a future-proof for transporting and storing heat materials. The paper concerns the current topic related to the miniaturization of cooling heat exchangers. There are many studies in the literature on the phase transition of refrigerants in the flow in pipe minichannels. However, there is a lack of studies devoted to the condensation process in a small volume on the surface of pipe minichannels. The authors proposed a design of a small heat exchanger with a shell-and-tube structure, where the refrigerant condenses on the outer surface of the pipe minichannels cooled from the inside with water. It is a response to the global trend of building highly efficient, miniaturized structures for cooling and air conditioning heat exchangers. Two future-proof, ecological replacements of the CFC refrigerants still present in the installations were used for the experimental research. These are low-pressure fluids HFE 7000 and HFE 7100. The tests were carried out in a wide range of changes in thermal-flow parameters: G = 20–700 kg·m^−^^2^s^−1^, q = 3000–60,000 W·m^−^^2^, t_s_ = 40–80 °C.

## 1. Introduction

In recent years, there has been an increase in interest in heat transfer in small spaces, both in everyday life, as well as in the economy, technology, and science. The continuous increase in the power of energy devices is accompanied by the process of their size reduction and weight loss or the so-called downsizing, i.e., replacing a larger system with a smaller one (e.g., with a smaller capacity or smaller size) with the same parameters maintaining their previous performance (e.g., efficiency or performance). Such a procedure also places new demands on heat exchangers. An increase in heat transfer intensity is increasingly desirable. This applies to both single-phase media heat exchangers and two- and multi-phase systems.

In practice, there are various ways to intensify heat transfer. They may concern the very structure of the exchanger, including the selection of appropriate materials for the construction of walls through which the heat is exchanged, developing and modifying the heat exchange surface, and appropriate selection of the components of the exchanger. It is also important to select the appropriate working media and their thermal and flow parameters. The miniaturization of the flow channels also contributes to a significant intensification of heat transfer, where the reduction of the hydraulic diameter is accompanied by an increase in heat transfer coefficients. There are many works devoted to scientific research related to this.

In the early 20th century, Nusselt [1] investigated the heat transfer coefficient of film-wise condensations on a single tube. Since then, many authors have developed his theories.

Belghazi et al. [2] investigated condensation heat transfer of a pure fluid and binary mixture outside a bundle of smooth horizontal tubes. The comparison of experimental data with the modified condensation curve method showed good agreement (±10%). Ji et al. [3,4] analyzed the R134a, R1234ze(E), and R290 condensation process outside single horizontal titanium, cupronickel (B10 and B30), stainless steel, and copper tubes. The authors’ own correlation agreed well with experimental data.

Kang et al. [5] provided an investigation on the influence of the geometrical and thermal-hydraulic parameters of the heat exchanger on heat transfer focused on the steam condensation on the outer surface of a vertical tube in the presence of a non-condensable gas. The authors found that an increase in the air mass fraction results in a decrease of the mass flux of steam and the HTC. An increase in the wall subcooling causes a decrease in the HTC, due to the accumulation of air near the condensing surface. In paper by Ribeiro et al. [6], the authors provided a macroscopic performance analysis of a real compact heat exchanger to investigate the performance enhancements obtained by the use of turbulators. Li et al. [7] investigated the effects of inclination and flow velocity on steam condensation consisting of air on tube bundle external surfaces. The influence of geometric parameters, height, length, and the effect of outside angle in corrugated fins was investigated by Gholami et al. [8]. Jian et al. [9] analyzed the effects of the geometric parameters on the condensation process in the shell side of spiral wound heat exchangers. It was found that the lower tubes dominate the overall heat transfer of the test section, with the variations in the overall HTC. The authors found an optimal winding angle (for these conditions, 8°) for spiral wound heat exchangers used as a condenser, in which the condensation HTC is at a maximum value. Barz et al. [10,11] investigated experimentally the phase transition behavior of three commercial paraffins filled in a compact plate-fin heat exchanger. A theoretical modeling was conducted to predict the thermo-hydraulic performance of a compact printed circuit heat exchanger with semicircular straight channels by Sarmiento et al. [12]. The influence of surface enhancement of heat exchanger minichannels on heat transfer was investigated by [13,14]. Ozturk et al. [15] investigated experimentally a flat tube with minichannel compact heat exchangers with offset strip fins by non-uniform uninterrupted fin length. A thermo-hydraulic analysis of a compact heat exchanger for a simple recuperated supercritical CO_2_ Brayton cycle was conducted by Pandey et al. [16]. Experimental investigation and modeling of steam-heated supercritical CO_2_ compact cross-flow heat exchangers was conducted by Theologou et al. [17]. Compact heat exchangers with flat and corrugated structures have been analyzed numerically by Khan et al. [18]. Overall heat transfer coefficient, effectiveness, and thermal-hydraulic parameters were analyzed. Buonomo et al. [19] numerically investigated the thermal and fluid dynamic performance parameters in aluminum foam compact heat exchangers. The influence of the addition of nanofluids on heat transfer was investigated in [20,21]. In papers [22,23,24,25,26,27], the authors investigated the influence of refrigerant condensation on heat exchangers’ thermal performance. During the heat exchange taking place in the heat exchangers, a different cooling medium is often used as the medium to receive/transmit the heat flux. 

Minko et al. [28] analyzed forced convection condensation of steam on smooth horizontal tubes and tube bundles in the presence of non-condensables. Kang et al. [29] investigated steam condensation on the tube bundle in presence of non-condensable gas under free convection. Gu et al. [30] provided a wide analysis of convective condensation heat transfer for moist air on a three-dimensional finned tube. Jivani et al. [31] experimentally investigated Marangoni condensation of steam–ethanol mixtures on a horizontal smooth tube. Mauro et al. [32] provided a wide review on a flow pattern, condensation, and boiling inside and outside smooth and enhanced surfaces of propane (R290).

Liu et al. [33] analyzed theoretically laminar film condensation inside and outside vertical diverging/converging small channels. Asokan et al. [34] examined the thermal performance of a compact heat exchanger with mono and hybrid nanofluids containing Al_2_O_3_ and CuO nanoparticles as a heat exchange medium. Hoseinzadeh et al. carried out a series of works on the intensification of heat transfer in mini- and microchannel heat exchangers [35,36,37].

The presented literature review shows that there are many studies on the condensation of refrigerants during the flow in pipe minichannels. However, there are only few studies devoted to the refrigerants’ condensation process in a small volume on the surface of pipe minichannels. The authors proposed a design for a small volume shell-and-tube heat exchanger in which the refrigerant condenses on the outer surface of pipe minichannels cooled from the inside by water. It is a response to the global trend of building highly efficient, miniaturized heat exchanger structures for refrigeration and air conditioning installations.

## 2. Experimental Investigations

**Figure 1:**
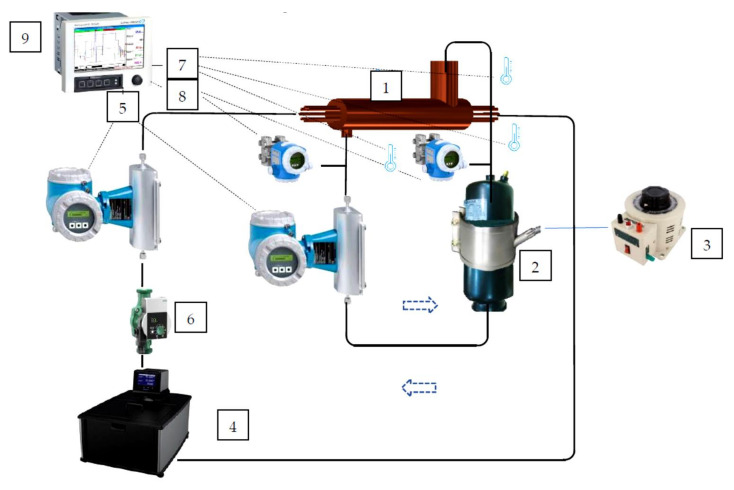
Pictorial diagram of the measuring stand: 1—heat exchanger; 2—refrigerant tank; 3—autotransformer; 4—cryostat; 5—mass flow meter; 6—circulation pump; 7—K-type thermocouple; 8—pressure sensor; 9—recording device.

In the experimental studies, two future-oriented fluids were used as heat transfer media. The 3M™ Novec™ 7100 Engineered Fluid methoxy-nonafluorobutane (C_4_F_9_OCH_3_) with ODP = 0 and GWP = 320 is intended to replace ozone-depleting substances (ODSs) and compounds with high global warming potential (GWP). The 3M™ Novec™ 7000 Engineered Fluid, 1-methoxyheptafluoropropane, is a non-flammable, low global warming potential (GWP) heat transfer fluid capable of reaching −120 °C. Both fluids have low toxicity. The experimental facility built for the purpose of investigating the condensation process of environmentally friendly refrigerants in heat exchangers is shown in Figure 1. The facility consisted of two loops: the working liquid circuit (refrigerant) and the cooling circuit (water).

The working liquid circuit consisted of a refrigerant tank, a micanite-resistant heater, a Coriolis mass flow meter, two differential pressure sensors, and service valves. The cooling medium circuit consisted of a cryostat, in which the temperature of the liquid was regulated, a centrifugal pump with continuously adjustable capacity, a Coriolis mass flow meter, and control valves. In order to ensure an even distribution of the water mass flow rate through the individual cooling channels, the flow resistance was compared along the length of the channels. The discrepancies between the results for individual channels were within the range of ±2%. The temperature of both fluids was monitored with K-type thermocouples. Measurement data were recorded with a data recorder.

The subject of the tests on the stand was a shell-and-tube heat exchanger. The heat exchanger was made in accordance with the original design. It consisted of seven copper minichannels with an internal diameter d_i_ = 4 mm, body with internal diameter D_i_ = 30 mm, length L = 200 mm equal to the heat exchange length. In the tested exchanger, horizontal pipe channels with an outer diameter Di = 6 mm were used. It is a small diameter compared to those practically used in classic heat exchangers. According to Kandlikar’s [38] classification, ducts of this diameter should be classified as conventional. However, according to Mehendale [39], ducts of this diameter are included in compact heat exchangers (D_h_ = 1–6 mm). On the basis of this criterion, the diameter D_i_ = 6 mm can be classified as minichannels (compact heat exchangers).

**Figure 2:**
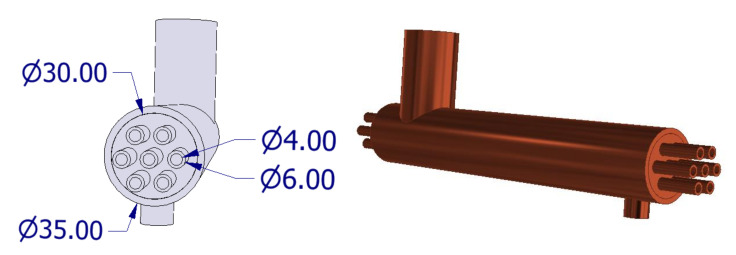
General view of the shell-and-tube exchanger.

The horizontal and vertical pitch of the tube bundle were P_t_ = 0.01 m and P_v_ = 0.01 m, respectively. The channels were arranged in a parallel configuration as shown in Figure 2. The exchanger was equipped with inlet connections and an outlet, which were, respectively, the supply and return of the exchanger with the refrigerant. The exchanger body has been sealed with two copper lids 2 mm thick with seven holes each. The holes with an inside diameter equal to the outside diameter of the cooling channels were arranged in parallel in three adjacent rows. All components of the exchanger were connected together by brazing.

After joining all the components, the exchanger was subjected to a pressure leakage test (0.35 MPa). After 24 h, no leakage was found. In order to minimize heat loss, the exchanger and its fittings have been thermally insulated with a synthetic rubber cover. The flow of HFE low-pressure refrigerant was forced by heating the refrigerant tank above the boiling point at a given pressure. As a result of heating, the medium evaporated with a certain intensity depending on the amount of thermal energy supplied to the interior of the tank. As a consequence, it was possible to adjust the flow rate of the medium during its circulation in the measuring system. After leaving the tank, saturated dry steam reached the inside of the heat exchanger through the inlet connector. Inside the exchanger body, the medium has condensed in volume on the surface of the cooling channels. The refrigerant condensate was discharged from the exchanger through an outlet connection. Thereafter, the refrigerant homogeneous fluid flowed through a Coriolis centrifugal flow meter to measure the refrigerant mass flow rate. The flow meter also acted as a density measuring instrument. The accuracy of the density and temperature measurement of this device was ±0.5 kg·m^−3^ and ±0.5 °C, respectively. Using the HART protocol of the flowmeter, it was possible to simultaneously record several measured values. A similar measurement solution was used for the cooling circuit. Here, the generator of movement was a centrifugal pump forcing cooling water to flow through the channels. The water flow rate was regulated by the rotation of the pump impeller, the main valve before the measuring section and by the intake valves of the cooling channels. The three-stage pump speed control and the cut-off valve adjustment ensured a constant supply of water with a certain intensity. The experimental research was conducted with due diligence and accuracy. Before starting the basic research, the experimental stand was subjected to verification tests. The accuracy of instruments and measuring apparatus was checked. The resistance to water flow inside each tube was measured. Comparing them with each other showed that they were within ±3%. This allowed for the conclusion that the water flow rate distribution will be uniform (within the measurement error). There is no inspection window in the outer jacket of the exchanger to observe the condensation structures. This was due to the small size of the exchanger and the fear of disrupting the condensation process. During the research it was assumed that the amount of refrigerant vapor was supplied to the exchanger in a sufficient amount to evenly fill its very small volume. Moreover, the water in the tubes was flowing at a sufficiently high speed that its temperature varied little along the length of the flow (less than 1 K). This resulted in a constant (almost constant) difference between the condensation temperature and the outer wall of the horizontal pipe, which contributed to the uniformity of the condensation process. The temperature of the outer wall of the pipe during condensation was determined on the basis of the knowledge of the cooling water temperature and the resistance to heat transfer from the water side and conduction in the pipe. This procedure was much more accurate than direct measurements, taking into account the small dimensions of the exchanger and the possibility of disturbances in the temperature field due to the introduction of sensors with finite dimensions. The task of the cryostat was to regulate the inlet water temperature and to cool it after heating the medium with steam during its flow through the measuring section. As already mentioned, the tested shell-and-tube mini heat exchanger contained seven straight-axis parallel channels on which the process of film condensation of the refrigerant took place. The design of the exchanger made it possible to include any number of tubes in the heat transfer process, from one to seven. This was performed by passing the cooling medium (water) through selected tubes that were to participate in the heat exchange. Such actions resulted in a change of the heat exchange surface, and this change in the parameters describing the process of film condensation on the heat exchange surface, e.g., mass flux density *G*, the flowing condensate velocity *w*, thickness of the condensate film *δ*, etc. The uncertainty of measuring devices is presented in Table 1.

The inlet valves upstream of the individual cooling channels were fitted to enable water to be supplied to any number and combination of cooling channels. The task of the cryostat was to regulate the inlet water temperature and to cool it after heating the medium with steam during its flow through the measuring section. The uncertainty of the measuring devices is presented in Table 1.

The heat transfer coefficient during the refrigerant condensation on horizontal tubes was determined from the dependence on the efficiency of a shell-and-tube mini heat exchanger:

(1) $$\dot{Q}=\frac{\pi \cdot n\cdot L\cdot \Delta t}{\frac{1}{\alpha_{w}\cdot d_{i}}+\frac{1}{2\lambda }\;\ln\frac{d_{e}}{d_{i}}+\frac{1}{\alpha_{\exp}\cdot d_{e}}}$$

where n—number of cooling channels, L—exchanger length, λ—heat conduction coefficient of the channel wall material (copper), d_i_—internal diameter of the minichannel, d_e_—external diameter of the minichannel, α_w_—heat transfer coefficient from the cooling water side, Δt—the difference in temperatures in the exchanger calculated as the difference between the saturation temperature of the refrigerant t_s_ and the average temperature of the cooling medium (water) t_f_.

Experimental studies of the condensation process were carried out while maintaining a constant saturation (condensation) temperature of the refrigerant at the level of about 32 °C. During the experimental tests, the changes in this temperature amounted to ±2 K. The change in the difference between the condensation temperature and the wall temperature of the pipe channel surface was caused by changing the temperature of the cooling water at the inlet to the exchanger. Therefore, it can be concluded that the presented values of the heat transfer coefficient are given for a constant (almost constant) condensation temperature of the medium in the range of 32 (±2) °C. From Equation (1), the dependence on the heat transfer coefficient α_exp_ is obtained in the form:

(2) $$\alpha_{\exp}=\frac{1}{d_{e}\cdot \left(\frac{\pi \cdot n\cdot L\cdot \Delta t}{\dot{Q}}-\frac{1}{2\lambda }\;\ln\frac{d_{e}}{d_{i}}-\frac{1}{\alpha_{w}\cdot d_{i}}\right)}$$

The heat transfer coefficient from the cooling water side *α_w_* was calculated based on criterion equations of forced convection during laminar motion in channels:

(3) $$\alpha_{w}=\frac{{\mathrm{Nu}}_{f}\;\lambda }{d_{i}}$$

where Nu_f_ is a number describing the intensity of the heat transfer as cooling medium flows through a cylindrical horizontal channel. In line with the fact that the range of liquid Reynolds number is in the range from 3000 to 10,000, the Nusselt number was determined by Nu_f_ and was calculated according to the modified Hausen formula [40], the use of which is appropriate in the field of transient and turbulent flow, Re_f_ = 2300 ÷ 15,000. The equation is:

(4) Nuf=0.116·1+diL2/3·Ref−125·Prf1/3·μfμw0.14

where Re_f_ is the Reynolds number for fluid, Pr_f_—Prandtl number for fluid temperature in the core, μ_f_—dynamic viscosity for fluid, μ_w_—dynamic viscosity for fluid temperature at the wall.

The heat efficiency of the exchanger was determined from the formula

(5) $$\dot{Q}=\dot{m}\left(h_{v}-h_{l}\right)$$

where:

$\dot{m}$ is the condensate mass flow rate, h_v_—steam enthalpy on the inlet of the exchanger, h_l_—liquid enthalpy at the outlet of the exchanger.

The heat flux density was determined by Equation (6):

(6) $$q=\frac{\dot{Q}}{A}$$

where A—heat transfer surface of the exchanger (external surface of active tubes).

Conducting experimental research, balance calculations of the heat exchanger were made. The heat flux removed from the condensing medium $\dot{Q}$ (Equation (5)) was compared with the heat flux ${\dot{Q}}_{w}$ w taken by the cooling medium—water:

(7) $${\dot{Q}}_{w}={\dot{m}}_{w}c_{w}\left(t_{w1}-t_{w2}\right)$$

where:

${\dot{m}}_{w}$ is the mass flow rate of water cooling the condenser, $c_{w}$—specific heat of water, and $t_{w1}$, $t_{w2}$ are the water temperature at the inflow and outflow from the condenser, respectively.

The calculations show that the compliance of the values of both heat fluxes was within ±5%. This proves the high accuracy of the measurements carried out.

Experimental research of the process was carried out on the basis of the presented research methodology. The tests were carried out in the steady state in the following range of thermal-flow parameters (Table 2).

The following is a graphical interpretation of the test results for the refrigerants’ condensation in a shell-and-tube heat exchanger.

**Figure 3:**
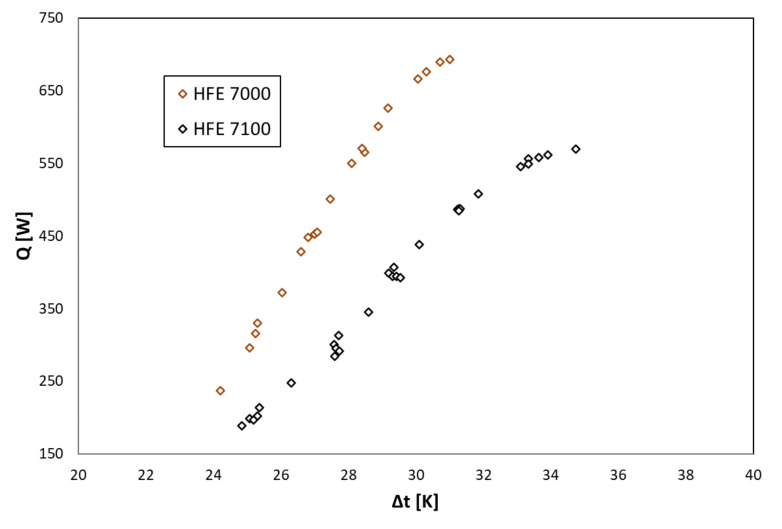
The dependence of the heat output of the shell-and-tube heat exchanger on the mean temperature difference of the refrigerant and the water cooling of the condenser, $\dot{Q}=f\left(\Delta t\right)$.

Figure 3 shows the dependence of the heat output of the tested exchanger on the mean temperature difference of the refrigerant and the water cooling of the condenser.

**Figure 4:**
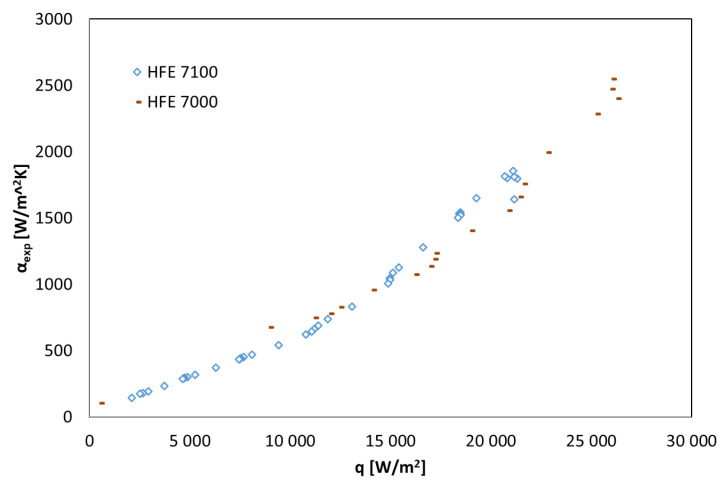
Results of experimental tests of thermal condensation of the refrigerants in the shell-and-tube exchanger, concerning the dependence of the heat transfer coefficient on the heat flux density, α_exp_ = f(q), HFE 7100 and HFE 7000 refrigerants.

**Figure 5:**
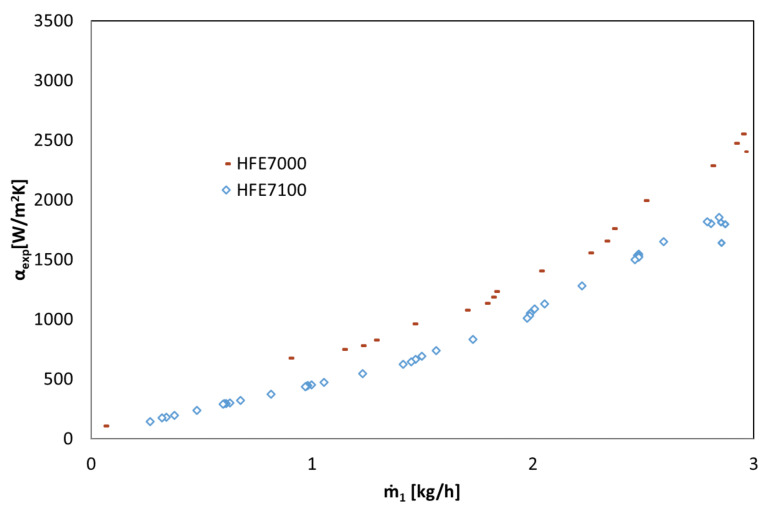
Thermal characteristics of the tested exchanger in the form of the dependence of the heat transfer coefficient on the mass flow rate α = f (ṁ_1_), refrigerant HFE 7100, HFE 7000.

**Figure 6:**
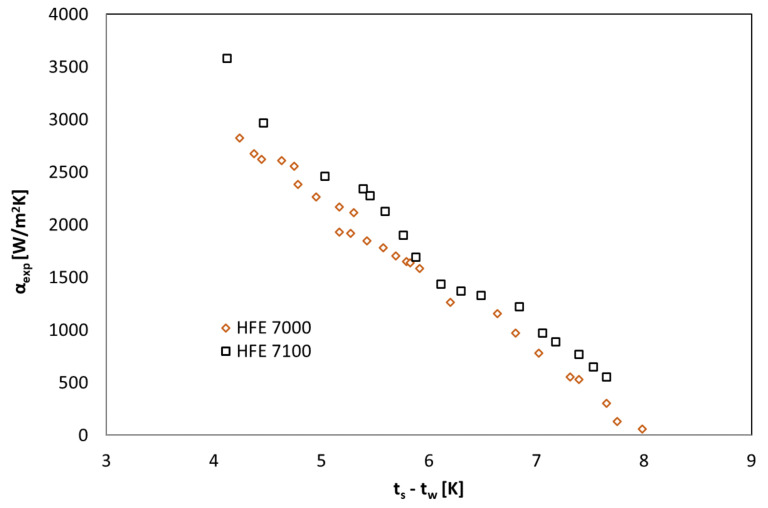
Thermal characteristics of the tested exchanger in the form of the dependence of the heat transfer coefficient on the difference between the saturation temperature and the external wall of the channel temperature α = f(t_s_ − t_w_), HFE 7100 and HFE 7000 refrigerants.

Figure 4, Figure 5 and Figure 6 show the thermal characteristics of the tested heat exchanger in the form of the dependence of the heat transfer coefficient on the heat flux density on the cooled surface, α_exp_ = f(q), the mass flow rate α_exp_ = f(ṁ), and the difference between saturation temperature and the outer wall of the channel temperature α = f(t_s_ − t_w_). All pipe channels participated in the heat transfer, n = 7. The presented characteristics for the two refrigerants HFE 7000 and HFE 7100 are very similar due to their similar thermal properties.

The heat-flow characteristics of the heat exchanger presented in Figure 4, Figure 5 and Figure 6 show that the value of the heat transfer coefficient during condensation on the surface of horizontal smooth pipes strongly depends on the heat flux density q on the cooled surface, which is related to the amount of the condensing medium described by the mass flow rate ṁ. The increase in heat flux density on the cooled surface causes an increase in the value of the heat transfer coefficient α. At the same time, the value of this coefficient decreases with the increase in the difference between saturation and wall temperatures, which results directly from Newton’s law for heat transfer with heat transfer on the wall.

**Figure 7:**
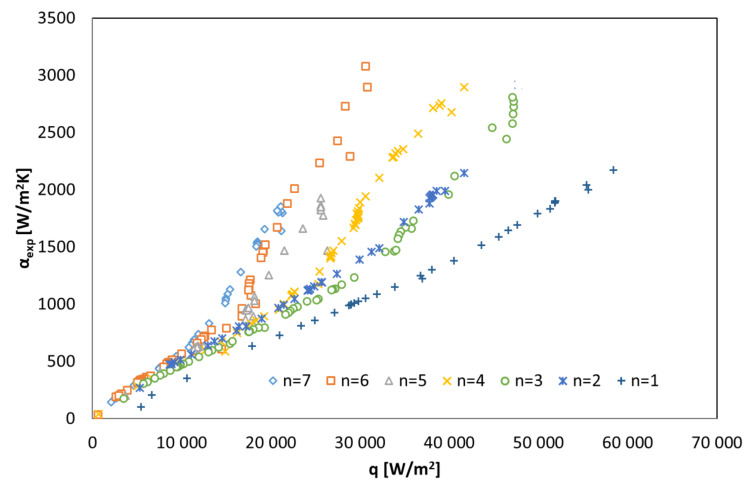
Collective experimental research data on the condensation of the HFE 7100 refrigerant in a shell-and-tube exchanger, concerning the dependence of the heat transfer coefficient on the heat flux density; n—number of active pipe minichannels.

Figure 7 shows the dependence of the heat transfer coefficient on the heat flux density on the exchanger surface with a different number of active cooling channels n for HFE 7100. The tests were carried out in the full range of changes in the thermal and flow parameters of the heat exchanger, activating successively an increasing number of channels through which the water flowed.

The data illustrated in Figure 7 show a clear dependence of the heat transfer coefficient on the heat flux density. The relationship is visible for all analyzed variants of the coolant flow (number of active channels varies within n = {1; 7}). The higher the value of the heat flux density, the greater the heat exchange intensity caused by higher values of the heat transfer coefficient α_exp_. The highest values of the heat transfer coefficient at q = const. have been noted at the maximum number of active cooling channels n. The reason is the increase in the heat exchange surface, which occurs when successive active channels are introduced into the condensation process. The increase of the heat exchange surface directly affects the reduction of the saturation temperature difference and the wall temperature (t_s_ − t_w_) and the decrease of the condensate thickness on the outer surface of the horizontal tube of the exchanger. This is due to the comparison of the formulas for Fourier’s law and Newton’s law for the boundary layer in the form of a condensate film. Hence:

(8) $$\alpha_{C}=\frac{\lambda \prime }{\delta }$$

**Figure 8:**
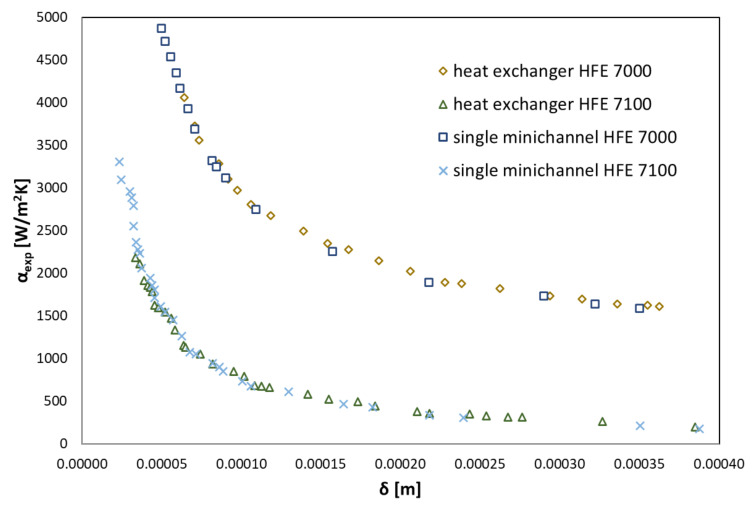
Experimental data of the dependence of the heat transfer coefficient on the condensate thickness of the HFE 7000 and HFE 7100 refrigerants.

From Equation (7), the thickness of the condensate (boundary layer) is determined, which inversely proportionally depends on the value of the heat transfer coefficient, as shown in Figure 8.

**Figure 9:**
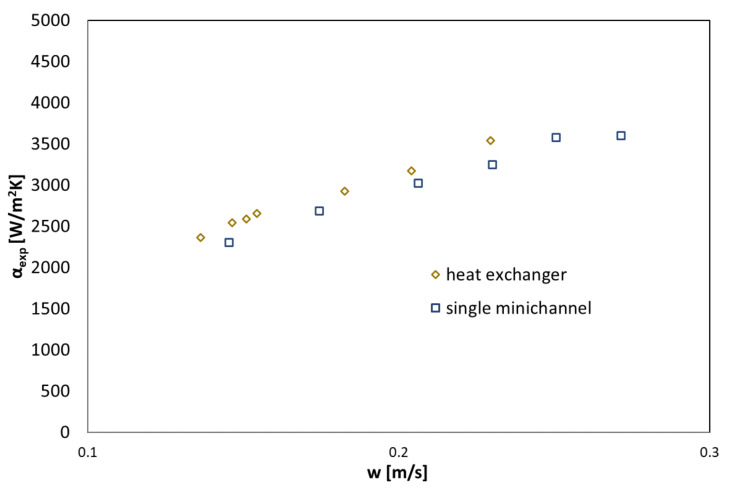
A comparison of experimental data of the heat transfer coefficient on the condensate velocity and condensate thickness during the HFE 7000 condensation process.

**Figure 10:**
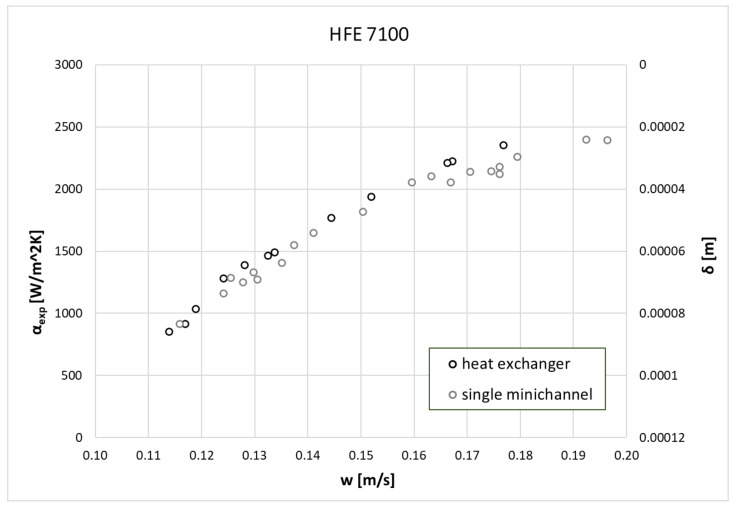
A comparison of experimental data of the heat transfer coefficient on the condensate velocity and condensate thickness during the HFE 7100 condensation process.

As can be seen, the heat transfer coefficient decreases with the increase of condensate thickness. Similar behavior was noted for both HFE 7000 and HFE 7100 refrigerants. Slightly higher HTC values were observed during HFE 7000 condensation. The familiarity of the thickness of the condensate makes it possible to determine the refrigerant mass flow density G and the condensate flow rate on the cooled wall. This is due to the dependence:

(9) $$G=\frac{\dot{m}}{2\;\delta \;L}$$

where δ is the thickness of refrigerant condensate on the external surface of heat exchanger’s channel, L is the minichannel’s length, w is the condensate velocity. The use of Equations (7) and (8) made it possible to develop the characteristics of the condensation process in the form of the common dependence of the heat transfer coefficient α and the thickness of the condensate film δ on the condensate velocity on the cooled wall of the channel. Figure 9 and Figure 10 show such dependencies for both tested refrigerants in the case of one active exchanger tube and all seven.

From the data presented in Figure 9 and Figure 10, it can be seen that the value of the heat transfer coefficient increases with the increase of the condensate velocity. The reason for this increase is the decrease in the thickness of the condensate film, which directly reduces the value of the thermal resistance between the condensing steam and the outer wall of the channel.

**Figure 11:**
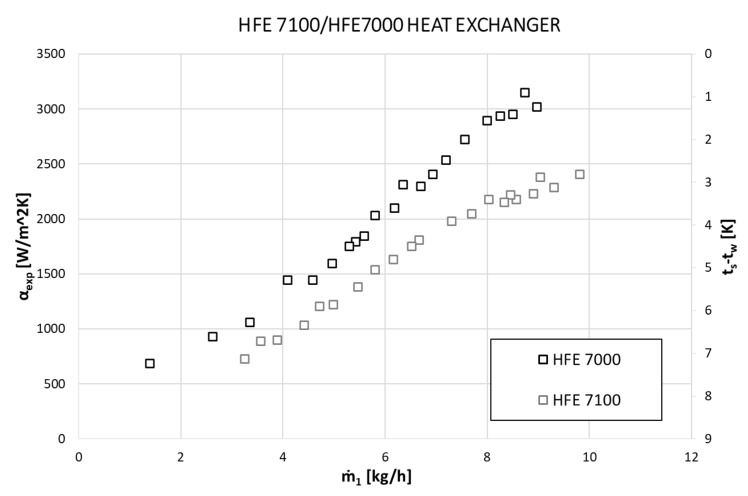
Experimental data on the dependence of the heat transfer coefficient α_exp_ and the difference between the saturation temperature of the refrigerant t_s_ and the wall temperature of the minichannel t_w_ on the mass flux flow of the refrigerant ṁ during the HFE 7000 and HFE 7100 refrigerants’ condensation.

Figure 11 shows the mutual comparison of the dependence of the heat transfer coefficient α and the value of the difference between the saturation temperature of the refrigerant t_s_ and the wall temperature of the minichannel t_w_ on the mass flow of the refrigerant per one active exchanger channel.

**Figure 12:**
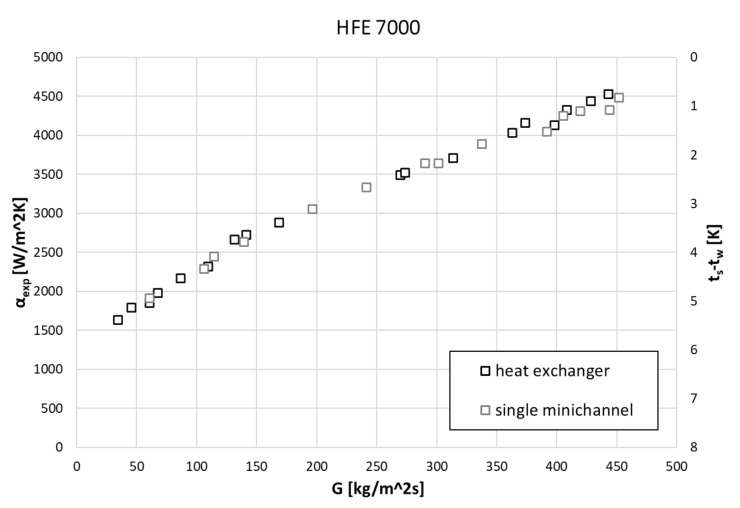
Experimental data for HFE 7000 in the form of correlation between the heat transfer coefficient α, mass flux density G, and temperature difference t_s_ − t_w_.

It is clearly visible that along with the increase in mass flux, the HTC values also increase, and at the same time, the difference in saturation and wall temperatures decreases. Similar behavior was observed during the condensation of both refrigerants. Figure 12 shows the dependencies for the HFE 7000 refrigerant concerning the heat transfer coefficient α, mass flux density G, and temperature difference t_s_ − t_w_. One is for the entire heat exchanger when all channels are active, the other is only for condensation on one active tube. The given dependencies show that the values of the heat transfer coefficient are comparable (or actually the same) in both cases. The extended analysis shows that this is met when all thermal and flow parameters are the same in both modes of the exchanger’s operation. This applies to the mass flux density G, the thickness of the condensate film δ, the difference between the saturation and wall temperatures, and the condensation temperature and pressure. The obtained convergence of the research results proves the correct conduct of the research process.

The results of the experimental tests were compared with the results of calculations according to the correlation of various authors, which are summarized in Table 3.

In order to verify the applicability of the models describing the heat transfer during condensation of refrigerants in a heat exchanger, the residual analysis method using MAPE (mean absolute percentage error) was used:

(17) $$\mathrm{MAPE}=\frac{1}{n}\sum_{i=1}^{n}\left[\frac{\left|{\mathrm{Nu}}_{\mathrm{th}}-{\mathrm{Nu}}_{\exp}\right|}{{\mathrm{Nu}}_{\exp}}\right]\cdot 100\%.$$

The mean absolute percentage error is shown in Table 4.

The results of the experimental tests were compared with the modeling results according to the dependencies presented in Table 3; significant discrepancies were found between the experimental data and the modeling results according to the correlation of other authors. The smallest discrepancies were found for the Shekriladze and Gomelauri [43] model (32.9%). MAPE for the Fujii and Oda [45] was 39.1%, Bohdal and Kruzel [42] model 57.4%, Ji et al. [2] model 59.5%, and Stephan and Preußer [41] model was 91.5%. The largest discrepancies were found for Kutateładze [44], where MAPE was 91.7%. The above discrepancies result from the fact that the correlations adopted for comparison are recommended for conventional channels [37,40] or flow inside minichannels [42]. Hence, they are of little use for calculating surface condensation in mini spaces.

Due to the lack of satisfactory convergence of the correlations available in the world literature describing condensation on the surface of the minichannels of a heat exchanger with experimental data, the authors of the article proposed their own, innovative model describing external heat transfer during the condensation of refrigerants in volume on the surface of horizontal smooth pipes of small diameter. The equation takes the form:

(18) $${\mathrm{Nu}}_{\mathrm{th}}=0.006{\;\mathrm{Ga}}^{-0.14}{\;\mathrm{Pr}}^{5.1}{\;K}^{0,8}$$

where Ga is Galileo number:

(19) $$\mathrm{Ga}=\frac{{g\;d}_{e}^{3}}{v^{\prime 2}}$$

and K is the condensation phase transmission describing number:


(20) $$K=\frac{r}{c_{p}{}^{\prime }\;\Delta T}$$

**Figure 13:**
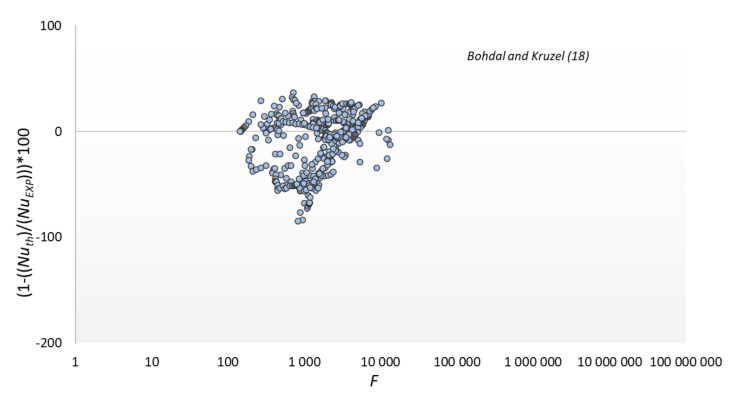
Comparison of experimental data to the model of Bohdal and Kruzel (18).

**Figure 14:**
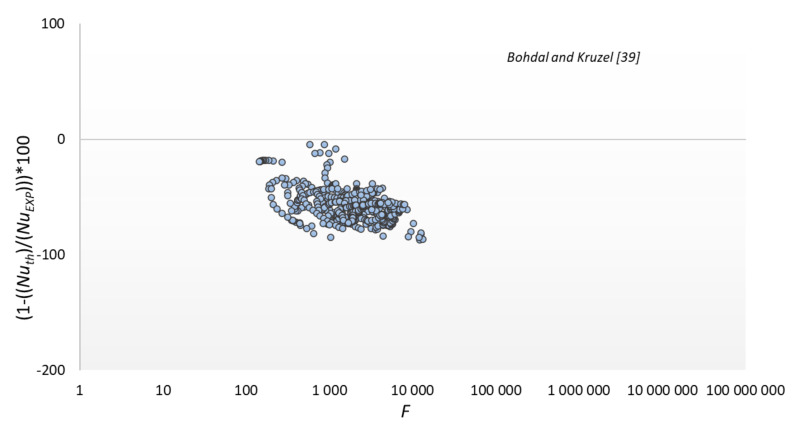
Comparison of experimental data to the model of Bohdal and Kruzel [42].

**Figure 15:**
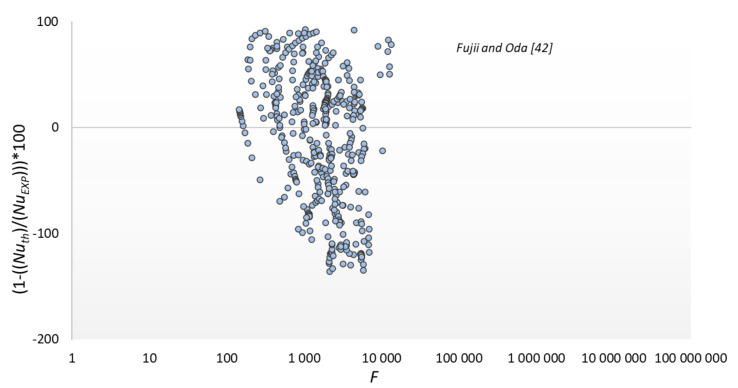
Comparison of experimental data to the model of Fujii and Oda [45].

**Figure 16:**
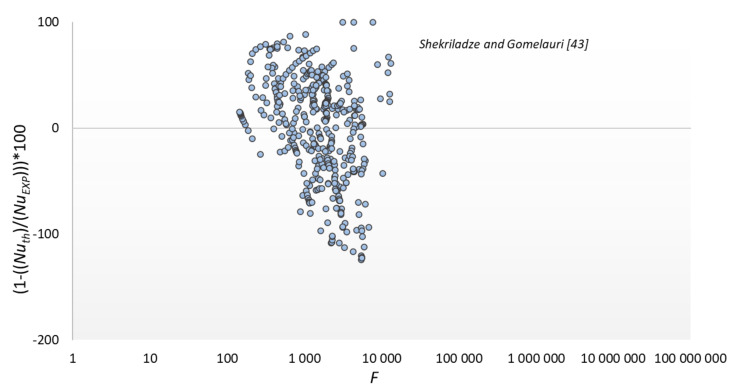
Comparison of experimental data to the model of Shekriladze and Gomelauri [43].

The results of comparing the experimental data with the modeling results according to the relationship of our own and some other authors are shown in Figure 13, Figure 14, Figure 15 and Figure 16. The graphs compare the mean percentage error with F, which is a dimensionless quantity describing the physical properties and thermal-flow parameters of the condensing refrigerants (14).

It can be seen at Figure 13 that the model shows the highest compliance with the results of experimental research. Mean absolute percentage error for Equation (18) is 16.2%, which is satisfactory for two-phase flow.

To sum up, it should be noted that this study presents research data in the field of condensation of ecological refrigerants in heat exchangers. During the tests, the values of the heat output of the exchanger operating in various thermal and flow conditions were determined. The original characteristics of the condensation process were presented in the form of dependencies describing the value of the heat transfer coefficient on the quantities describing the two-phase flow, including the mass flux density level G, temperature difference t_s_ − t_w_, and heat flux density q. The obtained data also made it possible to calculate the velocity w and the thickness of the condensate film δ formed on the cooled surface of the pipe minichannel. It has been shown that it is very important to properly select the operating parameters of the shell-and-tube heat exchanger. This applies in particular to the optimal use of the active heat exchange surface. Its size determines the value of the difference between the saturation temperature and the outer wall t_s_ − t_w_ of the channel that is generated automatically in the exchanger space. Introducing too much of the medium vapor to the exchanger in relation to the active heat exchange surface causes an increase in the temperature difference t_s_ − t_w_, which results in an increase in the amount of condensed liquid and an increase in the thickness of the condensate film δ. The increase in the thickness of the condensate film δ causes a decrease in the value of the heat transfer coefficient, which was confirmed by the conducted experimental studies.

## 3. Conclusions

1. Experimental studies were carried out on a heat exchanger with a shell-and-tube structure in which the refrigerant condenses on the outer surface of pipe minichannels cooled from the inside with water. Two future-proof, ecological substitutes for the CFC refrigerants still present in the installations were used for the experimental research. These are low-pressure fluids HFE 7000 and HFE 7100. The tests were carried out in a wide range of changes in thermal-flow parameters: G = 20–700 kg·m^−^^2^s^−1^, q = 3000–60,000 W·m^−^^2^, t_s_ = 40–60 °C.
2. The values of the thermal power of the exchanger operating under various thermal-flow conditions were determined, which were within the range $\dot{Q}$= 100–1500 W.
3. The original characteristics of the condensation process were presented in the form of dependencies describing the value of the heat transfer coefficient on the quantities describing the two-phase flow, including the mass flow density level G, temperature difference t_s_ − t_w_, and heat flux density q. The obtained test results also allowed the calculation of the velocity w and the thickness of the condensate film δ formed on the cooled surface of the pipe minichannel.
4. It has been shown that the correct selection of the operating parameters of the shell-and-tube heat exchanger is very important. This applies in particular to the optimal use of the active heat exchange surface. Its size determines the value of the difference between the saturation temperature and the outer wall of the t_s_ − t_w_ channel that is generated automatically in the exchanger space. Introducing too much of the medium vapor to the exchanger in relation to the active heat exchange surface causes an increase in the temperature difference t_s_ − t_w_, which results in an increase in the amount of condensed liquid and an increase in the thickness of the condensate film δ. The increase in the thickness of the condensate film causes a decrease in the value of the heat transfer coefficient.
5. The value of the heat transfer coefficient during the condensation of the refrigerant on the surface of the horizontal pipe minichannel increases with the increase of the heat flux density and the mass flux density of the flowing condensate.
6. The authors developed their own correlation to calculate the value of the heat transfer coefficient during the condensation of the refrigerant on the horizontal pipes of small diameter. It shows high agreement with the results of experimental research. The mean absolute percentage error is 16.2%, which is satisfactory for a two-phase flow. The correlation has been verified experimentally for refrigerants HFE 7000 and HFE 7100.

## Figures and Tables

**Table 1:** Measurement uncertainty of the equipment used to measure the refrigerant condensation process in the heat exchanger.

Measured Value	Device	Measuring Range	Max. Uncertainty
Mass flow	Coriolis effect mass flow meters	0–450 kg·h^−1^	±0.15%
Absolute pressure	Piezoresistive sensor	0–2500 kPa	±0.05%
	Differential manometer	0–50 kPa	±0.075%
Temperature	Thermocouple TP-201K-1B-100	−40–+475 °C	±0.2 K

**Table 2:** The scope of experiment carried out during HFE 7000 and HFE 7100 refrigerants’ external condensation in shell-and-tube heat exchangers.

The Measured Variable	Unit	Range of Parameter Changes
Mass flow rate $\dot{m}$	kg·h^−1^	1–15
Mass flux density level G	kg·m^−^^2^s^−1^	20–700
Heat flux $\dot{Q}$	W	100–1500
Heat flux density q	W·m^−^^2^	3000–60,000
Saturation temperature t_s_	°C	40–80

**Table 3:** Correlations used to compare the results of own experimental research for heat transfer during refrigerants’ external condensation.

Author	Correlation
Ji et al. [3]	$h_{p}=0.728{\left(\frac{{\mathrm{rg}\lambda }_{l}^{3}\rho_{l}^{2}}{\mu_{l}d_{0}\left(t_{s}-t_{w}\right)}\right)}^{1/4}=0.656{\left(\frac{{\mathrm{rg}\lambda }_{l}^{3}\rho_{l}^{2}}{\mu_{l}d_{0}q}\right)}^{1/3}$ (10)
((10(Stephan and Preußer [41]	Nu=4.364+0.0861/L*1.331+0.1PrDe Re/L0.83 (11) where Re, D_h_, L, and Pr are Reynolds number, hydraulic diameter, channel length, and Prandtl number, respectively, L^∗^ is the dimensionless thermal input length L*=L/DeRe Pr
Bohdal and Kruzel [42]	Nux=0.38 Re0.81 Ku0.47 ρvρl−0.15 (12) where: Re=2·wv devl+vv and: $\mathrm{Ku}=\frac{{q\;\rho }_{l}}{{r\;\rho }_{v}\;G}$
Shekriladze and Gomelauri [43]	${\mathrm{NuRe}}_{\mathrm{tp}}^{-1/2}=\frac{0.9+0.728F^{\frac{1}{2}}}{{\left(1+3.44F^{\frac{1}{2}}+F\right)}^{\frac{1}{4}}}$ (13) where: $F=\frac{{\mu h}_{\mathrm{fg}}\mathrm{gd}}{{\mathrm{kU}}_{\infty }^{2}\cdot T}$ (14) and: Retp=ρU∞dμ
Kutateładze [44]	${\mathrm{Nu}}_{l}=0.16{\;\mathrm{Pr}}^{\frac{1}{3}}+\frac{100-62{\;\mathrm{Pr}}^{\frac{1}{3}}}{X}$ (15)
Fujii and Oda [45]	$\mathrm{Nu}={\left({\mathrm{Nu}}_{\mathrm{gr}}^{4}+{\mathrm{Nu}}_{\mathrm{sh}}^{4}\right)}^{\frac{1}{4}}$ (16) where ${\mathrm{Nu}}_{\mathrm{gr}}={\mathrm{Nu}}_{\mathrm{Nu}}N^{-S}$ Nush=0.91+G−113Retp,mv1/2N−0.14 where: $G=\frac{k\Delta T}{{\mu h}_{\mathrm{fg}}}\left[\frac{\mu \rho }{\rho_{v}\mu_{v}}\right]$

**Table 4:** Comparison of the results of experimental research and calculations according to the correlations of various authors in the form of mean absolute percentage errors (MAPE) for heat exchange during HFE 7000 and HFE 7100 refrigerants’ external condensation in heat exchanger.

Author	MAPE [%]
Ji et al. [2] Stephan and Preußer [41]	59.5 91.5
Bohdal and Kruzel [42]	57.4
Shekriladze and Gomelauri [46] Kutateładze [44] Fujii and Oda [45]	32.9 91.7 39.1

## Data Availability

Data sharing is not applicable to this article.

## Nomenclature

A	area (m^2^)
d	diameter (m)
G	mass flux density (kg·m^−2^·s^−1^);
	dimensionless quantity
h	enthalpy (J·k^−1^)
L	length (m)
ṁ	mass flow rate (kg·h^−1^)
Nu	Nusselt number
q	heat flux density (W·m^−2^)
Q	heat flux (W)
r	heat of condensation/evaporation (J·k^−1^)
Re	Reynolds number
t	temperature (^o^C)
T	temperature (K)
U	vapor velocity
w	velocity (m·s^−1^)
Index	
1	per one channel
c	condensation
exp	experimental
e	external
f	fluid
gr	gravity
h	hydraulic
H	hydrostatic
i	internal
l	liquid
sh	shear stress
th	theoretical
t	total
tp	two-phase
v	vapor
w	wall, water
∞	free stream
Greek symbols	
α	heat transfer coefficient (W·m^−2^∙K^−1^)
Δ	difference
λ	thermal conductivity (W·m^−1^·K^−1^)
ρ	condensate film thickness (m)
ν	kinematic viscosity (m^−2^s^−1^)
Acronyms	
HE	heat exchanger
HTC	heat transfer coefficient
